# Pancreas-specific CHRM3 activation causes pancreatitis in mice

**DOI:** 10.1172/jci.insight.132585

**Published:** 2021-09-08

**Authors:** Jianhua Wan, Jiale Wang, Larry E. Wagner, Oliver H. Wang, Fu Gui, Jiaxiang Chen, Xiaohui Zhu, Ashley N. Haddock, Brandy H. Edenfield, Brian Haight, Debabrata Mukhopadhyay, Ying Wang, David I. Yule, Yan Bi, Baoan Ji

**Affiliations:** 1 Department of Cancer Biology, Mayo Clinic, Jacksonville, Florida, USA.; 2 Department of Gastroenterology, The First Affiliated Hospital of Nanchang University, Nanchang, PR China.; 3 Department of Pharmacology and Physiology, University of Rochester, Rochester, New York, USA.; 4 Prodo Laboratories Inc., Aliso Viejo, California, USA.; 5 Department of Biochemistry and Molecular Biology, Mayo Clinic, Jacksonville, Florida, USA.; 6 Department of Cardiovascular Medicine, Mayo Clinic, Rochester, Minnesota, USA.; 7 Department of Gastroenterology and Hepatology, Mayo Clinic, Jacksonville, Florida, USA.

**Keywords:** Gastroenterology, Inflammation, G protein–coupled receptors

## Abstract

Hyperstimulation of the cholecystokinin 1 receptor (CCK1R), a G protein–coupled receptor (GPCR), in pancreatic acinar cells is commonly used to induce pancreatitis in rodents. Human pancreatic acinar cells lack CCK1R but express cholinergic receptor muscarinic 3 (M3R), another GPCR. To test whether M3R activation is involved in pancreatitis, a mutant M3R was conditionally expressed in pancreatic acinar cells in mice. This mutant receptor loses responsiveness to its native ligand, acetylcholine, but can be activated by an inert small molecule, clozapine-*N*-oxide (CNO). Intracellular calcium and amylase were elicited by CNO in pancreatic acinar cells isolated from mutant M3R mice but not WT mice. Similarly, acute pancreatitis (AP) could be induced by a single injection of CNO in the transgenic mice but not WT mice. Compared with the cerulein-induced AP, CNO caused more widespread acinar cell death and inflammation. Furthermore, chronic pancreatitis developed at 4 weeks after 3 episodes of CNO-induced AP. In contrast, in mice with 3 recurrent episodes of cerulein-included AP, pancreas histology was restored in 4 weeks. Furthermore, the M3R antagonist ameliorated the severity of cerulein-induced AP in WT mice. We conclude that M3R activation can cause the pathogenesis of pancreatitis. This model may provide an alternative approach for pancreatitis research.

## Graphical abstract

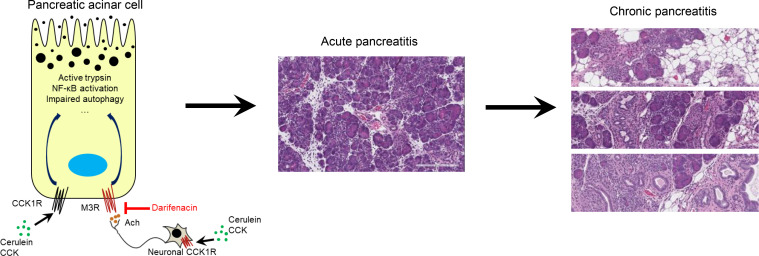

## Introduction

Pancreatitis, in both acute and chronic forms, is an inflammatory disease of the pancreas, which is painful and often lethal in severe cases (1). So far there is no proven pharmacological therapy to treat acute pancreatitis (AP). Supportive care with intravenous fluids, bowel rest, and pain management are the mainstays of therapy. The management of chronic pancreatitis (CP) is extremely challenging, and most patients remain symptomatic with pain, maldigestion, diabetes, and an increased risk of developing pancreatic cancer. There are no effective treatments to stop the progression or reverse these syndromes (1). Investigation into the pathogenesis and treatment of pancreatitis in humans faces many obstacles because of its deep anatomical location in the human body. Most of our knowledge on pancreatitis is based on research conducted using experimental animal models. In recent years, various experimental pancreatitis models have been developed in mice, rats, dogs, cats, guinea pigs, pigs, and zebrafish (2, 3). Yet, no effective, targeted drug for pancreatitis therapy has been approved by the US Food and Drug Administration (FDA), suggesting that there are difficulties in translating animal research into clinical practice. Alternative clinically relevant animal models need to be established for the investigation into the mechanisms of pancreatitis pathogenesis and the development of effective preventive and therapeutic interventions.

Currently, the most widely utilized experimental pancreatitis model is induced by hyperstimulation of the pancreas with cerulein, a cholecystokinin (CCK) analog, in rats and mice (2). This hyperstimulation results in AP by stimulating the CCK1 receptor (CCK1R), a G protein–coupled receptor located on pancreatic acinar cells, thereby activating trypsinogen and inflammatory signaling pathways (3). Many repetitive bouts of AP induction elicit chronic inflammatory changes in the pancreas (4), mimicking some features of CP. However, as we and others have previously shown, there are no functional CCK receptors in human pancreatic acinar cells, calling into question the relevance of cerulein-induced pancreatitis mouse models to the human disease (5–7). Although human acinar cells lack CCK receptors, the cholinergic muscarinic receptor 3 (CHRM3, or M3R) is highly expressed in human pancreatic acinar cells (5). However, the roles of M3R in pancreatic acinar cells in the pathogenesis of pancreatitis are unclear.

Muscarinic acetylcholine receptors, a group of G protein–coupled receptors (GPCRs), function in both the peripheral and central nervous systems, mediating innervation to visceral organs. Muscarinic receptors are further classified into the M1–M5 subtypes; among them the M1, M3, and M5 receptors are coupled with Gq proteins, while the M2 and M4 receptors are coupled with Gi proteins (8, 9). M3R is expressed in human pancreatic acinar cells (5). Both CCK1R and M3R belong to the GPCRs, and the activation of these receptors causes similar biological effects on isolated pancreatic acinar cells, including those involved in the calcium and PKC signaling pathways, amylase secretion, morphological changes, and lactate dehydrogenase release (5, 10, 11). The in vivo evidence came from the observation that exposure to potent cholinergic agonists, as seen with scorpion bites or organophosphate intoxication, is linked to the development of human AP (12, 13). Atropine, a cholinergic receptor antagonist, ameliorates pancreatitis induced by the combination of alcohol and cerulein, suggesting that cholinergic pathways are involved in pancreatitis in vivo (14). However, none of the evidence directly addresses whether the M3R on pancreatic acinar cells is important in the pathogenesis of pancreatitis. Scorpion venom contains multiple neurotoxins, and both nicotinic and muscarinic acetylcholine receptors are widely expressed throughout the central nervous system, the ganglionic neurons, the muscles, the mucosa, etc. Because using conventional pharmacological methods to study the specific roles that acinar M3R plays in the pathogenesis of pancreatitis has proved nonspecific, measures to activate the M3R specifically on pancreatic acinar cells should be developed.

In this study, we used a genetically modified mouse model to specifically activate M3R in pancreatic acinar cells in vivo. M3R stimulation in pancreatic acinar cells initiated AP, which progressed to CP. More importantly, the M3R antagonist ameliorated the severity of cerulein-induced AP. The data indicate that M3R activation is implicated in the development of both AP and CP. This model may provide an approach to discover preventive and therapeutic strategies for human pancreatitis.

## Results

### A clozapine-N-oxide–responsive DREADD mutant M3R mimics the functions of WT M3R.

Both CCK receptor and M3R belong to the GPCR family and elicit similar calcium signaling and amylase secretion patterns (15). Stimulation of CCK1R causes pancreatitis in rodents. However, it is unclear whether the stimulation of M receptors, located in pancreatic acinar cells, is involved in pancreatitis in vivo. To specifically activate the M3R in mouse pancreatic acinar cells, we decided to use a genetic mouse model with the conditional expression of a modified human M3 receptor. We used the chemical genetic approach to control the activation of receptors, with the designer receptors being exclusively activated by designer drugs (DREADD) (16). The DREADD mutant M3R (hM3R) no longer responds to the endogenous ligand acetylcholine but can be activated by exogenous clozapine-*N*-oxide (CNO) (17). Pancreas-specific expression of hM3R can be used to test the functionality of M3R activation without causing systemic side effects.

**Figure 1:**
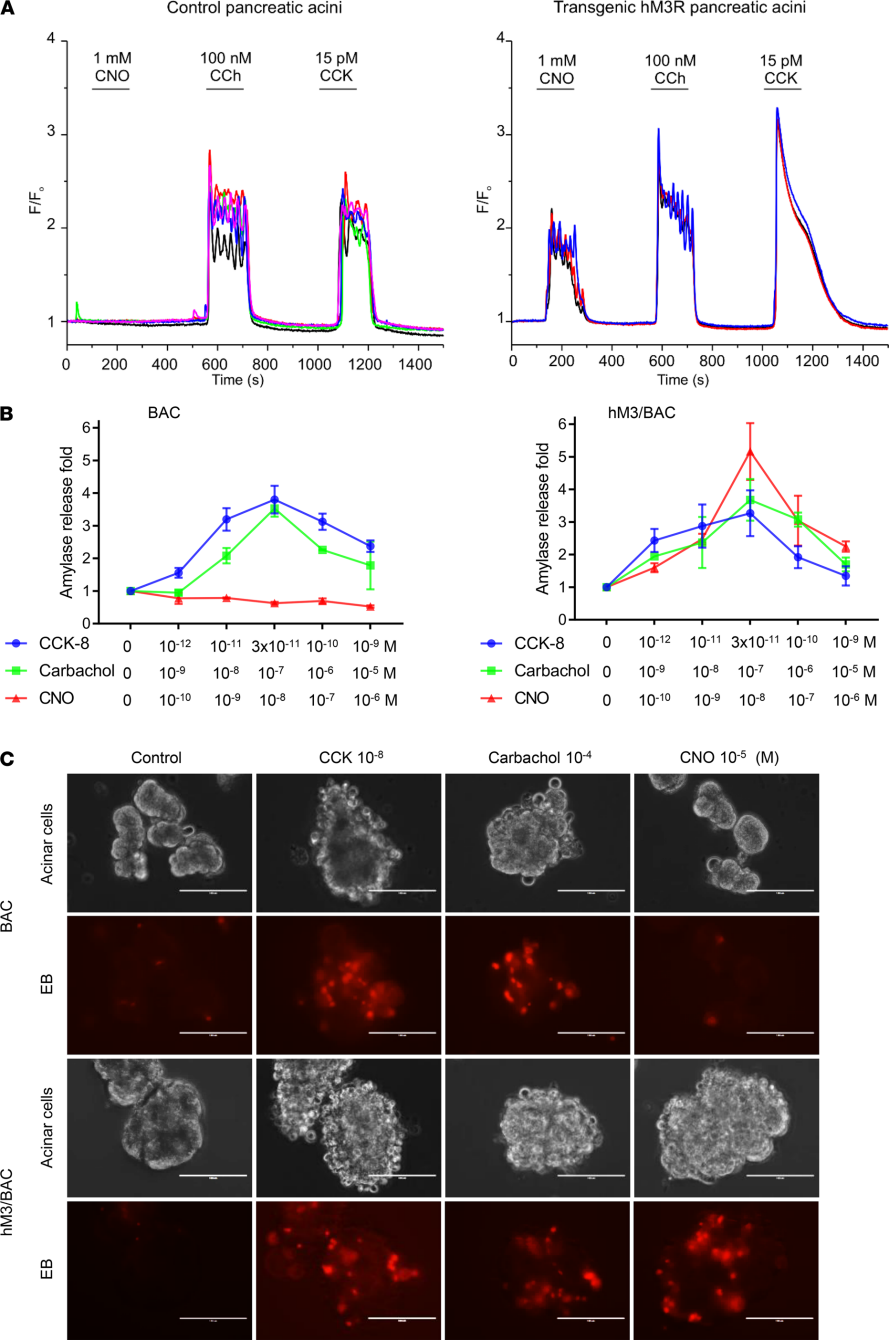
A mutant CNO-responsive hM3R mimics the functions of WT M3R in the pancreatic acinar cells of transgenic mice. (**A**) Intra-acinar calcium signals were recorded in fura-2–loaded pancreatic acinar cells isolated from control (left) and hM3R transgenic mice. CNO only elicited calcium signaling in acini expressing hM3R. The measurement was repeated in 5 mice each. (**B**) CNO dose-dependently induced a biphasic amylase secretion in acinar cells from hM3R transgenes (hM3R/BAC). Mean ± SEM (*n* = 3). Amylase secretion was comparable among CCK-, carbachol-, or CNO-stimulated acinar cells from hM3R/BAC mice (*P* 0.05). (**C**) Isolated primary pancreatic acinar cells were stimulated by saline (control), CCK, carbachol, or CNO. Cell membrane damages were manifested as blebbing and increased permeability to ethidium bromide (red). Note CNO caused damages only in pancreatic acini prepared from hM3R/BAC mice. Mean ± SEM (*n* = 3). Scale bar: 100 μm.

To achieve the conditional pancreatic acinar cell–specific expression of hM3R, R26-LSL-hM3R (*Loxp*-Stop-*Loxp*-hM3R) mice were crossed with BAC-Ela-Cre^ERT^ (BAC) mice, which expressed Cre^ERT^ driven by the pancreatic acinar cell–specific elastase I promoter (18) (hM3/BAC, Supplemental Figure 1A; supplemental material available online with this article; https://doi.org/10.1172/jci.insight.132585DS1). In these mice, the expression of hM3R alleles can be induced by administration of tamoxifen. The protein expression of hM3R was verified by Western blotting (Supplemental Figure 1B). The M3R mRNA expression levels in the transgenic pancreas were quantified by quantitative reverse transcription PCR and compared with expression levels found in endogenous M receptors in mouse pancreas (Supplemental Figure 1C) and human donor pancreas (Supplemental Figure 1D). Intra–acinar cell calcium release is a classic signaling event for both M3R and CCK1R and is purported to cause pancreatitis. Initially, we studied the calcium signaling in response to CCK, carbachol (CCh, an M receptor agonist), and CNO stimulations in freshly isolated mouse pancreatic acinar cells. Intracellular [Ca^2+^] signals were recorded in pancreatic acinar cells loaded with fura-2. Acinar cells from control mice were not responsive to the superfusion of CNO but responded to both CCh and CCK. In contrast, the acinar cells isolated from mutant hM3R-transgenic mice responded to CNO at similar levels compared to their response to CCh and CCK as measured by increasing intracellular [Ca^2+^] (Figure 1A).

In rodent pancreatic acinar cells, it is well-known that both CCK and CCh elicit biphasic amylase secretion in a dose-dependent manner and, at high concentrations, cause acinar cell damage. To test the functionality of mutant hM3R, both the amylase secretion pattern and cell damage were measured in pancreatic acinar cells isolated from the control and hM3R-transgenic mice. As expected, CNO stimulated a biphasic amylase release and acinar damage, visualized by blebbing and ethidium bromide staining, only in pancreatic acinar cells with transgenic hM3R expression (Figure 1, B and C). No response to CNO was observed in the control pancreatic acinar cells. Collectively, these data indicate that mutant hM3R functionally mimics M3R but only responds to CNO. Pancreas-specific transgenic expression of mutant hM3R can be used to study the role of M3R activation in pancreatitis in vivo.

### CNO induced the development of AP in hM3R-transgenic mice.

**Figure 2:**
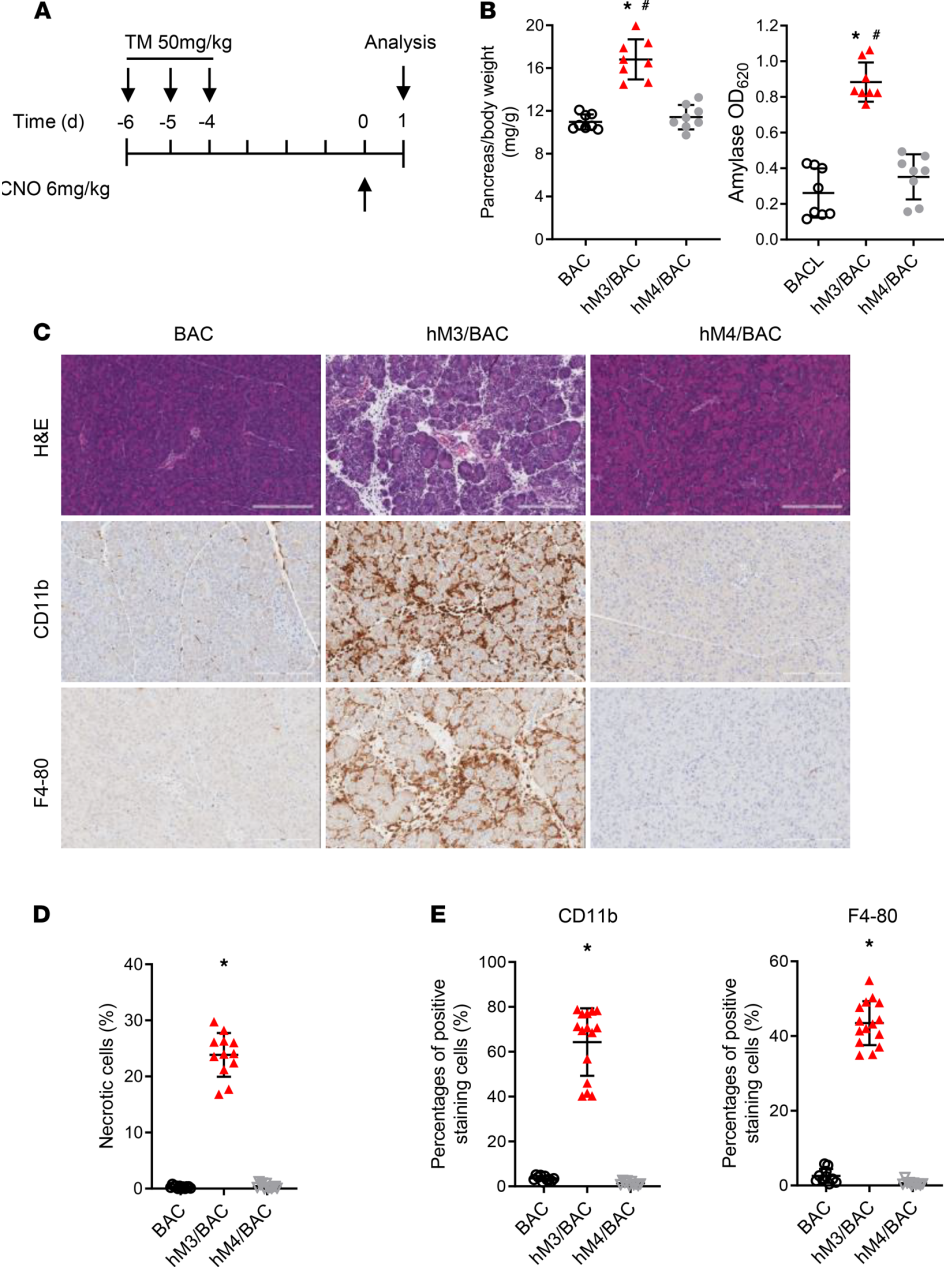
Acute pancreatitis was induced by CNO in hM3/BAC mice. (**A**) Tamoxifen (50 mg/kg; oral gavage) was administered on1e week prior to CNO (6 mg/kg; ip). Mice were sacrificed 24 hours after the CNO injection. (**B**) Pancreas edema (pancreas/body weight ratio, mg/g) and serum amylase were measured (*n* ≥ 8 mice/group). Mean ± SEM (*n* = 8). (**C**) HE staining and immunohistochemical (IHC) analysis of pancreatic tissues (scale bar: 200 μm). (**D**) Quantification of the necrotic cells. Mean ± SEM (*n* = 8). (**E**) Quantification of the inflammatory cells. Mean ± SEM (*n* = 8). *P 0.05, hM3/BAC vs. BAC group. ^#^*P* 0.05, hM3/BAC group vs. hM4/BAC group. Two-way ANOVA with Tukey’s test.

To study the effects of M3R activation on pancreatic acinar cells in vivo, hM3R expression was induced with tamoxifen followed by CNO administration to hM3/BAC mice. Controls including BAC mice and hM4R (a DREADD mutant Gi protein–coupled muscarinic 4 receptor) mice received similar treatments. Mice were sacrificed 24 hours after the initial dose of CNO, and the severity of pancreatitis was examined (Figure 2A). Pancreatic edema and serum amylase increased in the CNO-treated hM3R mice but not in the control mice or the hM4R mice (Figure 2B). Compared with the control mice, the hM3/BAC mice given CNO displayed dramatic inflammatory cell infiltration (Figure 2, C and E) and pancreatic acinar necrosis (Figure 2, C and D).

**Figure 3:**
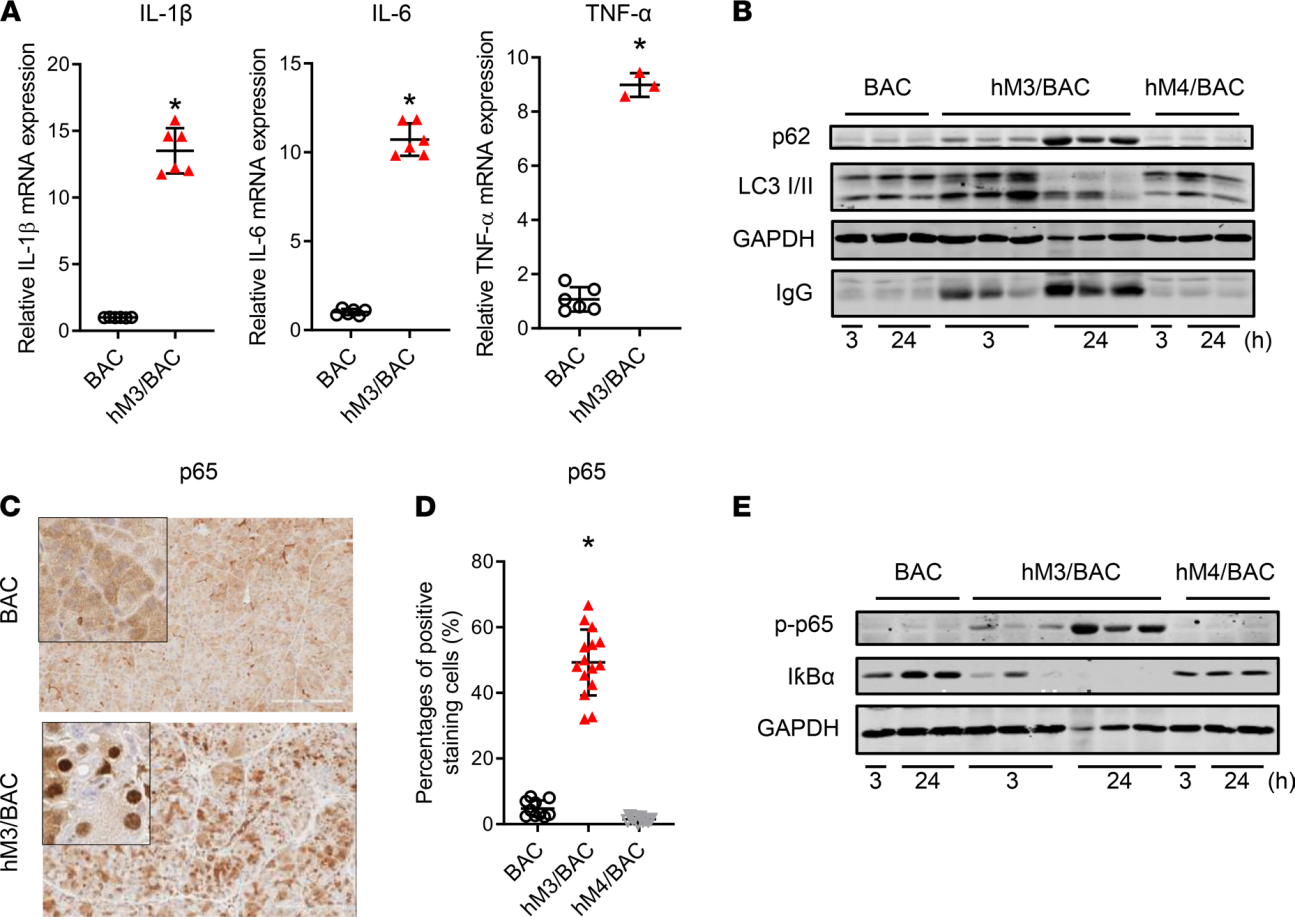
Activation of inflammatory signaling pathways in AP of hM3/BAC mice. (**A**) The mRNA expressions of the inflammatory factors in the pancreas were detected by quantitative reverse transcription PCR. Mean ± SEM (*n* = 6). (**B**) Autophagy flux was measured by Western blot (WB). Representative blots from 3 independent assays are shown. (**C**) The p65 nuclear translocation was detected by IHC (scale bar: 200 μm; original magnification of insets, ×200). (**D**) Quantification of the p65 nuclear–positive cells. Mean ± SEM (*n* = 6). (**E**) p65/RelA phosphorylation and IκBα degradation were detected by WB. Representative blots from 3 independent assays are shown. *P 0.05, hM3/BAC vs. BAC group. Two-tailed unpaired Student’s *t* test.

The mRNA expressions of proinflammatory factors, such as IL-1β, IL-6, and TNF-α, in the pancreas were found to be at dramatically increased levels in this AP model (Figure 3A). Impaired autophagy has been gradually recognized as one of the mechanisms of pathogenesis for pancreatitis (19, 20). In this model of pancreatitis, we also observed an accumulation of p62, a signaling scaffold and chaperone for poly-ubiquitinated proteins, and of LC3-II, a surface lysosomal marker, as the typical changes of impaired autophagy in the AP model of hM3R mice (Figure 3B). Proinflammatory NF-κB is also commonly activated in both human and experimental mouse pancreatitis (21). In the transgenic hM3R mice, CNO treatment increased p65/RelA nuclear translocation (Figure 3, C and D), as well as p65 phosphorylation and IκBα degradation (Figure 3E). These data indicated the enhanced activation of the proinflammatory NF-κB signaling pathway in the AP model of hM3R mice (21).

We further explored how altering the dose and injection times of CNO would affect the severity of AP (Supplemental Figures 2 and 3). With a single dose of CNO injection, the severity of pancreatitis reached its peak at the dose of 6 mg/kg (Supplemental Figure 2). With multiple injections repeated hourly (Supplemental Figure 3), the levels of AP were comparable between 5 injections at a lower dose (2 mg/kg) or 1 injection at the dose of 6 mg/kg. Repeated injections at the dose of 6 mg/kg did not further exacerbate pancreatitis (Supplemental Figure 3).

### CNO-induced AP exhibited more cell death compared with cerulein-induced pancreatitis.

**Figure 4:**
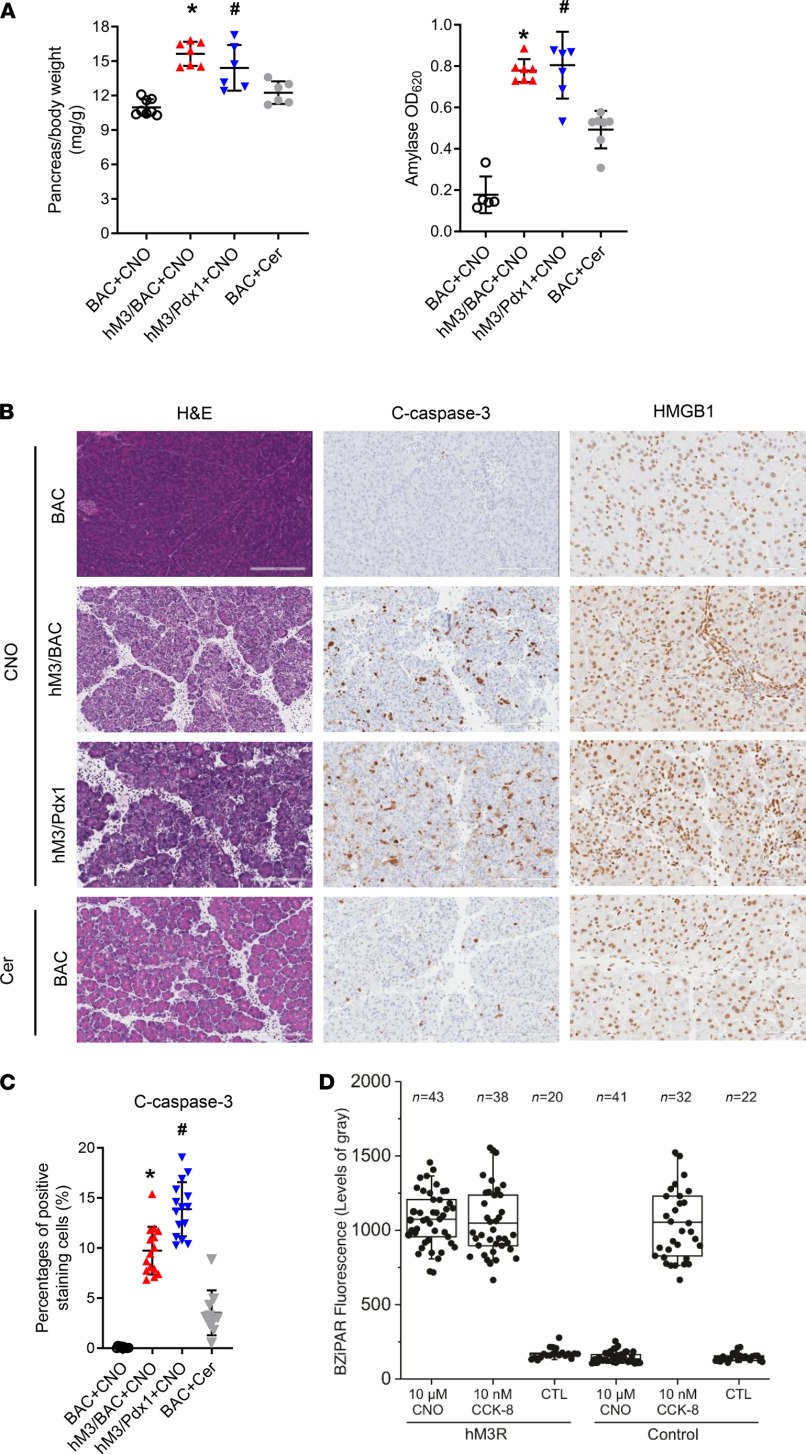
CNO induced more severe AP than cerulein. (**A**) Mice were injected with a single dose of CNO (6 mg/kg, ip) or 8 times of cerulein (100 μg/kg, ip) following tamoxifen treatment. Pancreatic edema and serum amylase were measured and compared. hM3R/Pdx1-Cre (hM3/Pdx1) double transgenic mice were included for comparison. Mean ± SEM (*n* = 6–8). (**B**) HE, IHC for cleaved caspase-3 (apoptosis), and HMGB1 (necrosis) staining of pancreatic tissues (scale bar: 200 μm). (**C**) Quantification of cleaved caspase-3 was calculated using ImageScope software. Mean ± SEM (*n* = 6–8). *P 0.05, hM3/BAC+CNO group vs. BAC+Cer group. ^#^*P* 0.05, hM3/Pdx1+CNO group vs. BAC+Cer group. Two-way ANOVA with Tukey’s test. (**D**) Trypsinogen activation was measured by BZiPAR fluorescence in control or acini expressing mutant hM3R. The box plots depict the minimum and maximum values (whiskers), the upper and lower quartiles, and the median. The length of the box represents the interquartile range.

Both BAC-Ela-Cre (exocrine pancreas only) and pdx1-Cre (both exocrine and endocrine pancreas) are widely used for pancreas-specific genetic manipulations. M3R is expressed in both exocrine and endocrine pancreas (22). Therefore, we also bred the LSL-hM3R mice with the pancreas-specific Pdx1-Cre mice (hM3R/Pdx1-Cre, hM3/Pdx1) to express hM3R in the pancreas. CNO caused a similar level of acute pancreatic injury in these mice as it did in the hM3/BAC mice (Figure 4, A–C). Next the severity of AP in hM3/Pdx1 and hM3/BAC mice was compared with the commonly used cerulein-induced pancreatitis model. When compared with the CNO-treated BAC mice, the BAC mice that were given 8 hourly injections of cerulein (100 μg/kg/h) presented increased pancreatic edema and serum amylase. Compared with cerulein-induced AP, greater levels of pancreatic edema and serum amylase were observed in both mouse models expressing hM3R, following a single injection of CNO (6 mg/kg) (Figure 4A). Histological and immunohistological analyses revealed that there was more acinar cell death as well as an increased inflammatory cell infiltration in the hM3R models (Figure 4, B and C). Pancreatic damage and inflammation were diffused throughout the whole pancreas of the hM3R mice after CNO induction, whereas the damage induced by cerulein was mild and patchy (Figure 4B). Intra–pancreatic acinar cell trypsinogen activation is a key event for the initiation of pancreatitis. Using a trypsin substrate BZiPAR [Rhodamine 110, bis-(N-CBZ-L-isoleucyl-L-prolyl-L-arginine amide), dihydrochloride], we found that both CCK and CNO were capable of increasing BZiPAR fluorescence in hM3R acinar cells, but only CCK increased fluorescence in the control acini. The level of trypsin activity was comparable between CNO and CCK stimulation in hM3R mice (Figure 4D).

### Different progression courses in cerulein- and CNO-induced AP models.

**Figure 5:**
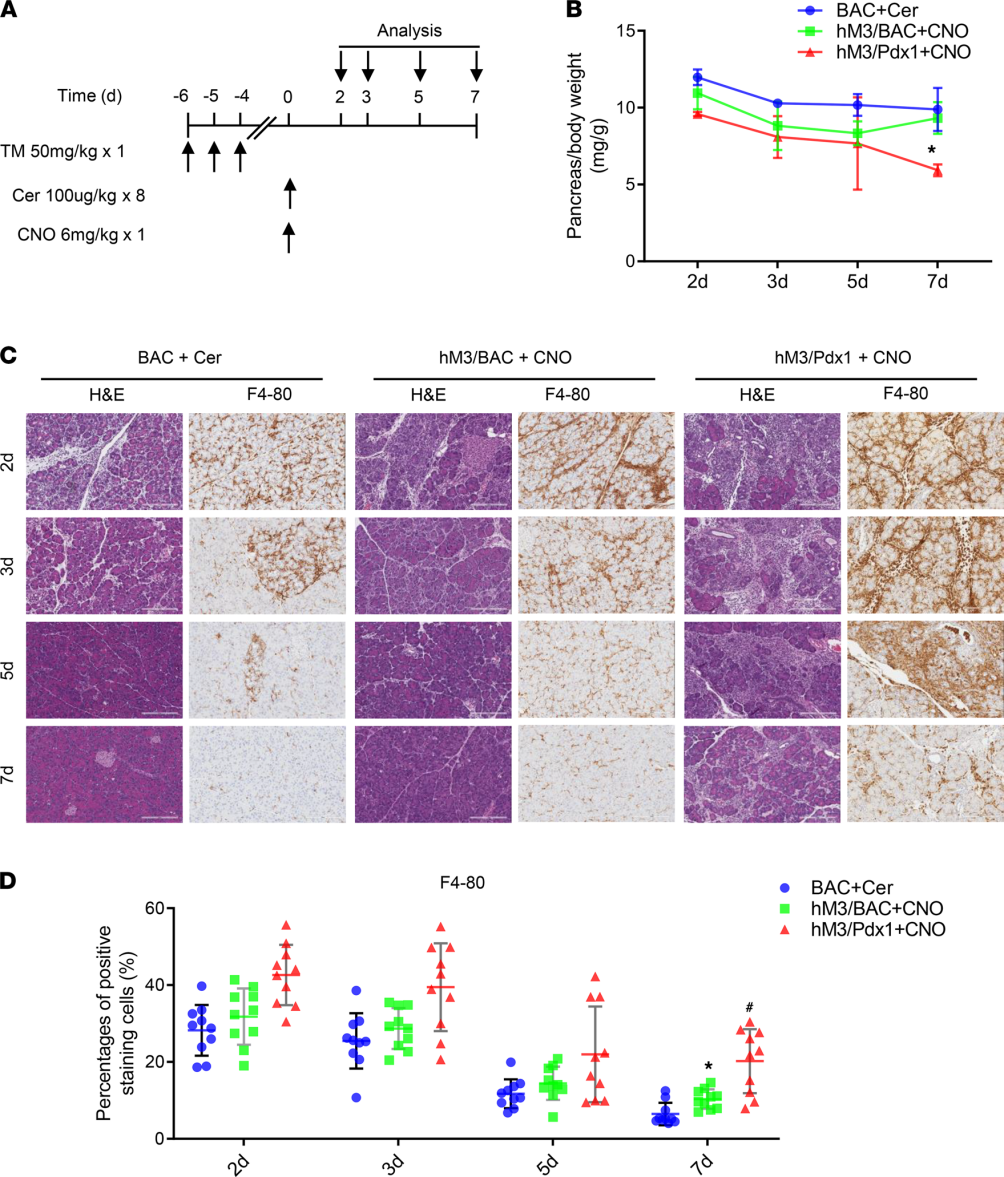
Pancreatitis recovery in cerulein-inducing BAC mice, CNO-inducing hM3/BAC, and hM3/Pdx1 mice. (**A**) Mice were injected with a single dose of CNO (6 mg/kg, ip) or 8 times of cerulein (100 μg/kg, ip) following tamoxifen treatment. (**B**) The pancreas shrinkage was measured following AP induction at indicated time points. Mean ± SEM (*n* = 3–5). *P 0.05, hM3/Pdx1+CNO group vs. BAC+Cer group. Two-way ANOVA with Tukey’s test. (**C**) Pancreas pathology was revealed by HE and F4-80 staining (scale bar: 200 μm). (**D**) Quantification of F4-80–positive cells. Mean ± SEM (*n* = 3–5). *P 0.05, hM3/BAC+CNO group vs. BAC+Cer group. ^#^*P* 0.05, hM3/Pdx1+CNO group vs. BAC+Cer group. Two-way ANOVA with Tukey’s test.

Next, we investigated the outcomes of acute pancreatitis after 1 episode of induction (Figure 5A). It is well-known that cerulein-induced edematous AP is mild, and the pancreatic damage is resolved in 1 week (Figure 5). Even though acute inflammation and damages were more severe in hM3/BAC mice than in cerulein-included mice, the pancreatic damage of hM3/BAC mice appeared completely resolved at day 7 following a single injection of CNO (Figure 5, B and C), albeit the recovery process was slower than that of cerulein-induced AP. In contrast, the pancreatic size of hM3/Pdx1 mice after a single CNO-induced AP became significantly smaller at day 7 after CNO injection (Figure 5B). Histology showed signs of early CP, including acinar cell atrophy and inflammation (Figure 5, C and D). These findings were inconsistent with the observation that the severity of AP in hM3/BAC and hM3/Pdx1 mice was similar (Figure 4). One likely explanation is that stem cell damage in hM3/Pdx1 mice impaired the recovery process because Pdx1-Cre mediates recombination in whole parenchymal pancreas, including putative stem cells (23), while BAC-Ela-Cre^ERT^ mediates pancreatic acinar cell–specific gene recombination (18).

### Recurrent AP causes CP in hM3R mice.

**Figure 6:**
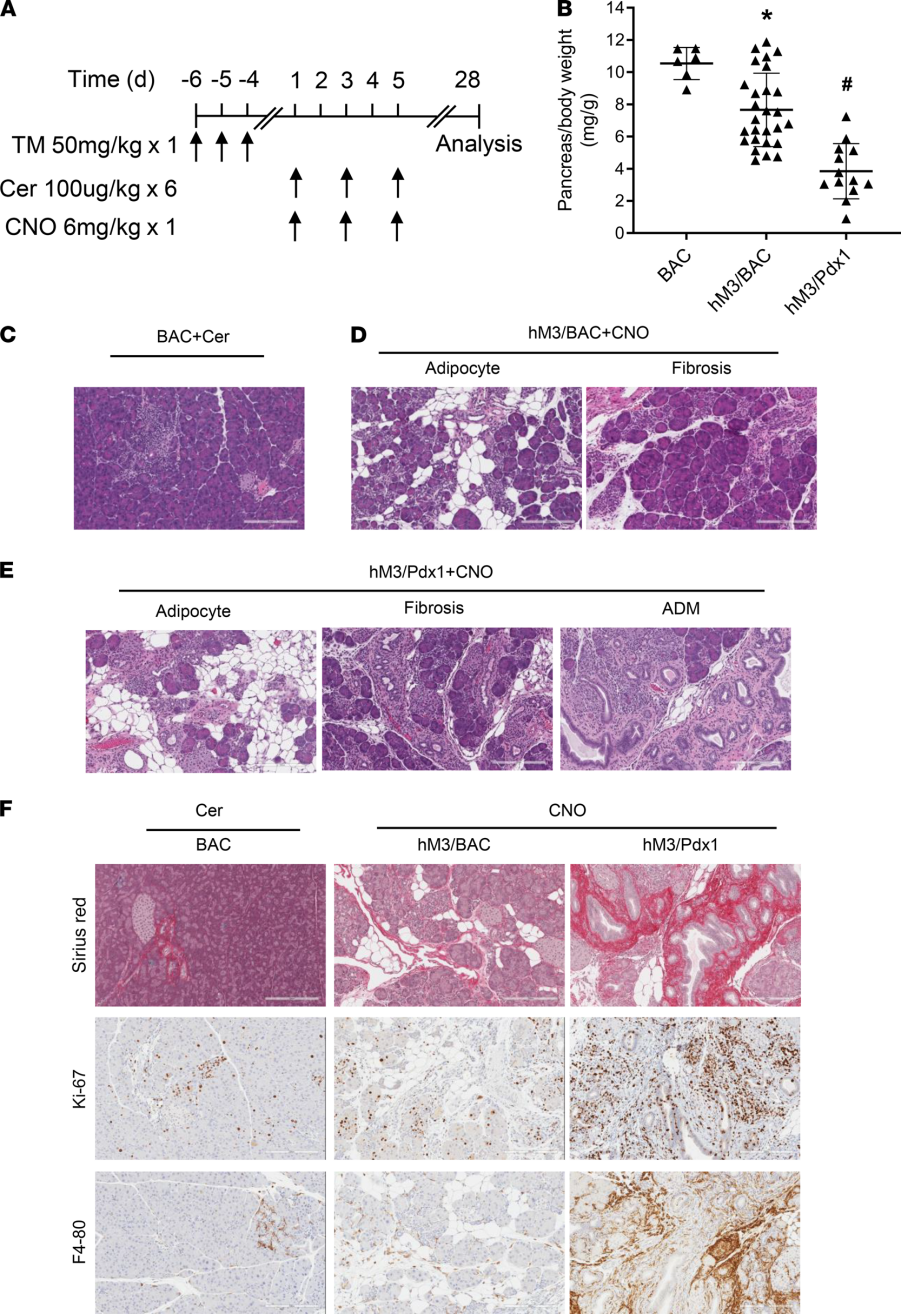
CP develops in hM3R mice. (**A**) Three episodes of acute pancreatitis were induced in BAC, hM3/BAC, hM3/Pdx1 mice. The pancreas tissues were harvested at 4 weeks following the initial pancreatitis induction. (**B**) Pancreas size was measured by pancreas/body weight ratio (mg/g). Mean ± SEM (*n* ≥ 6). (**C**) Representative HE staining in cerulein-induced mice. (**D**) Histology of CNO-induced CP in hM3/BAC mice. (**E**) In hM3/Pdx1 mice, more severe CP developed. (**F**) Fibrosis (Sirius red), cell proliferation (Ki-67), and chronic inflammation (F4/80) in BAC, hM3/BAC, and hM3/Pdx1 mice (scale bar: 200 μm). *P 0.05, hM3/BAC+CNO group vs. BAC+Cer group. ^#^*P* 0.05, hM3/Pdx1+CNO group vs. BAC+Cer group. Two-way ANOVA with Tukey’s test.

There is evidence that AP can progress to CP in both humans and mice (4, 24). Because the activation of the M3R in the pancreas caused a more severe bout of AP and the recovery process was slower than that of the cerulein model (Figure 5), we suspected that the hM3R mice were more susceptible to the development of CP following recurrent AP. To test this hypothesis, 3 bouts of AP were induced every other day by multiple cerulein injections in BAC mice or a single CNO injection in hM3/BAC and hM3/Pdx1 mice. Pancreatitis was evaluated 28 days after the first onset of AP (Figure 6A). The pancreas size in cerulein-treated mice returned to normal, consistent with the fact that CP rarely develops after short-term cerulein induction (2). In contrast, two-thirds of the pancreata from the hM3/BAC mice and all the pancreata from the hM3/Pdx1 mice became atrophic (Figure 6B). Notably, there were no differences in overall body weight among these groups (Supplemental Figure 4). Histologically, pancreata from cerulein-treated mice were nearly normal, showing few signs of focal acinar atrophy (Figure 6C). Two-thirds of the pancreata from hM3/BAC mice with smaller pancreas size showed typical features of CP, including chronic inflammation, fibrosis, adipose tissue infiltration, and pancreatic atrophy (Figure 6D). The chronic damage observed in the hM3/Pdx1 mice was more severe and manifested widespread chronic inflammation, fibrosis, adipose tissue infiltration, and pancreatic atrophy (Figure 6E). Acinar-ductal metaplasia (ADM) was commonly observed in the hM3/Pdx1 mice but rarely observed in the hM3/BAC or in the cerulein-induced BAC mice (Figure 6E). Meanwhile, Sirius red staining and immunohistochemistry staining with the antibodies of Ki-67 and F4/80 revealed prominent fibrosis, cell proliferations, and chronic inflammatory cell infiltrations in the hM3R mice, especially in the hM3/Pdx1 mice (Figure 6F). Taken together, CP developed in hM3R mice after 3 single doses of CNO injections.

### M3R antagonist alleviates cerulein-induced pancreatitis.

**Figure 7:**
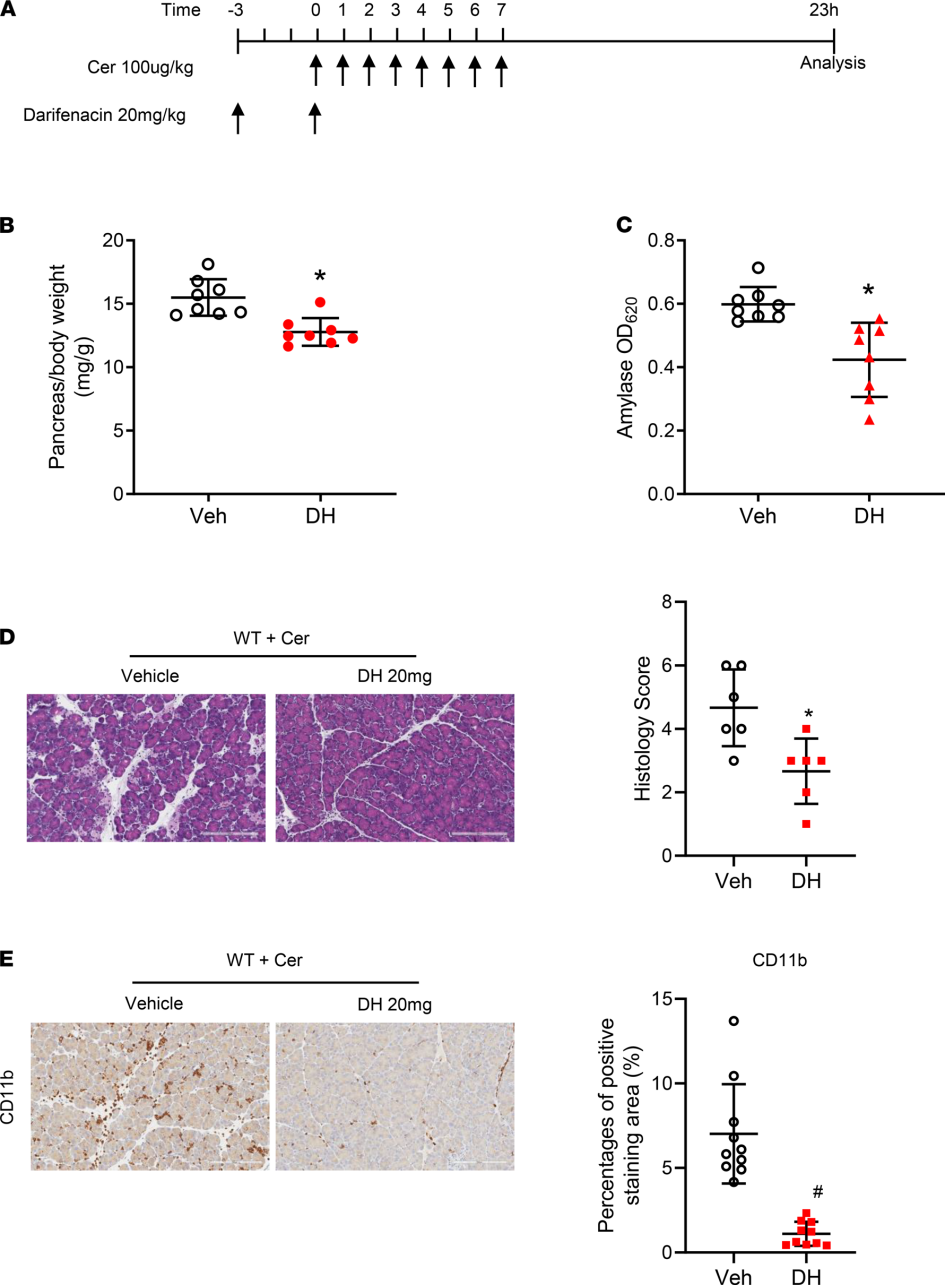
M3R-specific antagonist darifenacin ameliorated cerulein-induced AP. (**A**). Schema of treatment with darifenacin hydrobromide (DH) (20 mg/kg; ip), an M3R-specific antagonist. (**B**) DH treatment reduced cerulein-induced pancreas edema in C57/BL6 (WT) mice. (**C**) Serum amylase was lower in DH-treated mice (*n* = 8 mice/group) than saline-treated group. (**D**). DH treatment improved pancreatitis histology (scale bar: 200 μm). (**E**). CD11b inflammatory cells were reduced upon DH treatment (scale bar: 200 μm). *P 0.05 and ^#^*P* 0.01, DH group vs. vehicle group. Two-tailed unpaired Student’s *t* test.

Next, we wanted to determine whether the severity of pancreatitis would be improved by inhibiting the M3R in WT mice. For this purpose, we chose to use a selective M3R antagonist, darifenacin. Darifenacin (Enablex, Novartis) is an FDA-approved medication used to treat overactive bladder syndrome. It works by blocking the M3R in the bladder muscles and inhibiting bladder contractions, thereby decreasing the urgency to urinate (25). WT mice were pretreated with darifenacin (20 mg/kg) 3 hours prior to the initiation of cerulein-induced pancreatitis and were treated with a second dose of darifenacin after the induction of pancreatitis (Figure 7A). Treatment of darifenacin reduced pancreatic edema (Figure 7B) and serum amylase levels (Figure 7C). The features of pancreatitis histopathology (Figure 7D) and inflammatory cell infiltration (Figure 7E) were significantly improved compared with the histopathological and inflammatory features of the mice that had been pretreated with the vehicle. Notably, darifenacin had no direct effect on CCK1R on pancreatic acinar cells as measured by amylase secretion (Supplemental Figure 5). Collectively, these data support that acetylcholine release, likely due to CCK1R activation on neurons, is partially involved in the pathogenesis of cerulein-induced pancreatitis, and inhibition of the M3R may be beneficial to pancreatitis.

## Discussion

In this study, we demonstrated that pancreatic acinar cell–specific activation of the M3R in transgenic mice caused the development of both AP and CP. Selective inhibition of the M3R ameliorated the severity of pancreatitis. These findings indicate that M3R activation is involved in the development of both AP and CP. Targeting this receptor may be a preventive and therapeutic strategy for patients suffering from pancreatitis. When compared with the cerulein model of pancreatitis, this model is more severe and less patchy. Additionally, the induction of pancreatitis with CNO is less time-consuming.

Activation of the CCK1 receptor, a GPCR, in rodents by using cerulein, a CCK analog, is widely used in experimental pancreatitis studies. Hyperstimulation of the CCK1R in pancreatic acinar cells activated trypsinogen and proinflammatory signaling pathways, a key mechanism for the initiation of pancreatitis. However, whether the CCK1R exists on human pancreatic acinar cells has been controversial. We and several other groups have shown that human pancreatic acinar cells do not express the CCK1R and do not respond to CCK stimulations as measured by calcium signaling or amylase secretion assays (5–7, 26). In contrast, another group claimed they had observed CCK-elicited calcium signaling responses in acinar cells found in the human pancreas (27). However, in support of our previous findings, mRNA expression of the CCK1R was not detected by RNA-Seq in purified human pancreatic acinar cells extracted from healthy donors without exocrine pancreatic disorders. Instead, we found that the M3R was highly expressed in these human pancreatic acinar cells (Supplemental Figure 6) (Pei Wang, University of Texas Health San Antonio, San Antonio, Texas, USA, personal communication). Recent data from The Human Protein Atlas (https://www.proteinatlas.org/ENSG00000163394-CCKAR/tissue) also demonstrate that there are no CCK1Rs in the human pancreas (28). The Human Protein Atlas used tissues extracted from 95 human individuals to represent 27 tissues to determine the tissue specificity of all protein-coding genes, revealing that M3R was expressed and CCK1R was absent (28). In light of these data, we strongly believe that CCK1R is absent in normal human pancreatic acinar cells. Instead, the M3R is highly expressed in human pancreatic acinar cells (5).

M3R is classified in the GPCR family, which regulates the enzymatic secretion of many glands, including those found in the pancreas (29). The function of the M receptors in pancreatic acini has been studied in vitro (30, 31). Stimulation of the M receptors with CCh, an acetylcholine analog, causes effects such as peak-plateau calcium signaling, small G protein activation, biphasic amylase secretion, trypsin and NF-κB activation, acinar cell blebbing, and lactate dehydrogenase release. These effects are all similar to what was observed in CCK1R when stimulated with CCK (32). In mice, both M1R and M3R exist in pancreatic acinar cells, and likely both play a role in the mediation of these effects (31). The in vivo evidence of the M receptor’s involvement in pancreatitis in both humans and rodents largely stems from indirect studies (33). Human exposure to potent cholinergic agonists, such as scorpion bites or organophosphate intoxication (found in agricultural chemicals), can cause AP (34, 35). Lugea et al. reported that, in rats that were fed a diet of ethanol and further treated with cerulein, atropine significantly decreased pancreatic damage in alcohol-induced pancreatitis (14). Because both nicotinic and muscarinic acetylcholine receptors are widely expressed throughout the central nervous system, the ganglionic neurons, and the pancreas, it remains unclear whether the M receptors on pancreatic acinar cells are playing a role in the process of pancreatitis development.

In order to determine whether the direct activation of the M3 acetylcholine receptors located in pancreatic acinar cells can initiate the pancreatic inflammatory response in vivo, we utilized a genetically modified mouse model that expresses a version of the human M3R, specifically DREADD. These DREADD engineered GPCRs are used to precisely control the GPCR’s signaling pathways, such as Gq, Gs, and Gi (17). The conditional transgenic strains of mice, hM3R and hM4R, allow for Cre recombinase–mediated, restricted, tissue-specific expression of these pathway-selective DREADDs. These engineered receptors lose the ability to respond to the natural ligand acetylcholine but gain the ability to respond to a small synthetic molecule, CNO, with nanomolar potency (8). CNO is both pharmacologically inert and metabolically stable in mice, thus allowing us to use it in vivo to directly activate the hM3R and hM4R, respectively. For pancreatic acinar cell–specific expression, these mice were crossed with pancreas-specific Cre mouse lines. The mutant M3R expression level in transgenic pancreas was 4-fold of that in human pancreas. One possible explanation is that the R26 promoter used to express mutant M3R is stronger than the native M3R promoter. An alternative explanation is that the human samples from donors were not as fresh as snap-frozen mouse tissues. Nevertheless, the mutant M3R responses to CNO are functionally similar to endogenous M receptor responses to carbachol in terms of calcium signaling and amylase secretion in vitro. Upon CNO administration in vivo, we observed an upregulation of common signaling pathways in pancreatitis, including trypsin activation, impaired autophagy, NF-κB activation, and endoplasmic reticulum stress (36). These mice developed AP with pancreatic acinar cell death, edema, inflammatory cell infiltration, and elevated serum amylase levels. As a control, we used an hM4R mouse line to activate the Gi pathway in the pancreas. CNO stimulation of Gi failed to cause any detectable pancreatic damage. These findings support the hypothesis that the pathological pancreatitis changes were caused by Gq activation.

In this study, we used both BAC-based Ela-Cre^ERT^ transgenic mice (18), which mediate acinar cell–specific recombination, and Pdx1-Cre mice, which express Cre throughout the whole parenchymal pancreas from embryonic day E8.5 (37). We found that CP was more severe in mice with hM3/Pdx1-Cre. Since both BAC and Pdx1-Cre mice showed similar recombination efficiency (data not shown) and comparable levels of AP, recombination efficiency cannot be the cause of this phenomenon. However, because Pdx1-Cre recombination occurs in the whole pancreas, including in islet, acinar, and even pancreatic stem cells (38, 39), β cells’ malfunction results in higher insulin and reduced blood glucose levels, both of which may exacerbate pancreatitis (40). Additionally, the activation of the mutant M3 receptors may cause stem cell damages and therefore impair the recovery of hM3/Pdx1-Cre mice (37).

The edematous AP induced by cerulein is mild and recovered from within a few days. In contrast, the activation of hM3R by CNO caused more severe AP and CP to manifest after only 3 episodes of recurrent AP. Although several common signaling pathways in pancreatitis can be activated by both receptors, these data suggest that hM3R activates some yet-to-be-identified, unique damaging signaling pathways. The receptor-specific pathways will have important clinical implications for patient care and deserve more extensive investigation in future studies.

The cerulein method for inducing pancreatitis is the most used experimental model because of its ease of use and reproducibility. However, this model requires 8–12 hourly injections of cerulein, and the lesions are usually mild and patchy. Induction of CP using the cerulein method is more challenging, requiring weeks of repeated injections. In contrast, a single injection of CNO can initiate AP in hM3R mice. The damages are more diffuse than cerulein pancreatitis. Additionally, with only 3 rounds of AP using CNO, marked CP develops in the hM3R mice. Furthermore, the cost of CNO is much lower than cerulein. With this model, it is possible to induce reproducible AP and CP while saving more than 90% of the injection time and costs.

**Figure 8:**
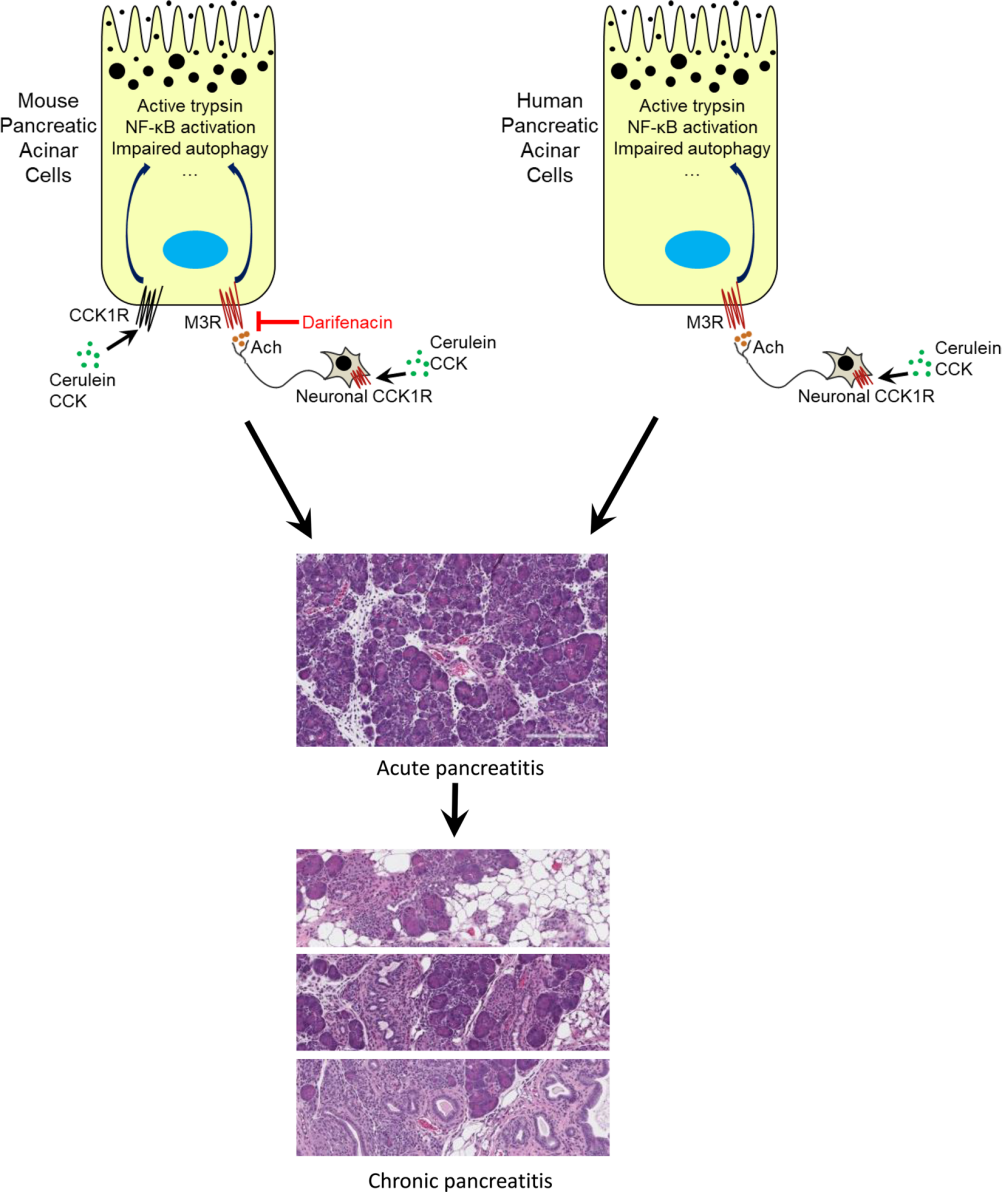
A proposed model: neuronal release of acetylcholine plays a role in the pathogenesis of pancreatitis. In rodents, cerulein activates CCK1Rs on both pancreatic acinar cells and neurons. Neuronal CCK1R activation mediates acetylcholine release, which subsequently stimulates muscarinic receptors on pancreatic acinar cells. Together, CCK1Rs and M receptors trigger pancreatitis. In contrast, on human pancreatic acinar cells there is no CCK1R. Neuronal secretion of acetylcholine initiates pancreatitis through M receptors located on pancreatic acinar cells.

It is well recognized that CCK, and its analog cerulein, cause pancreatitis by binding to the CCK1R in pancreatic acinar cells to activate trypsinogen and proinflammatory signaling pathways. It is striking that the M3R antagonist, darifenacin, was able to partially reduce the severity of pancreatitis in cerulein-induced pancreatitis in WT mice. The findings from Owyang’s group may provide a reasonable explanation. They established that CCK acts on vagal afferent cholinergic neuronal pathways to mediate pancreatic secretions in both rodent and human pancreata (41). Together with our findings here, it appears that both pancreatic secretions and pancreatitis elicited by CCK/cerulein are, at least partially, mediated by the CCK receptor located on neurons. Upon CCK/cerulein stimulation, the neuronal fibers release acetylcholine and activate M3R in pancreatic acinar cells to mediate secretion and inflammation. This revised working model, as shown in Figure 8, may be important in the pathogenesis of human pancreatitis since human pancreatic acinar cells do not express CCK receptors. Darifenacin (Enablex) is already an FDA-approved drug that is currently being used to treat overactive bladders with symptoms of urinary incontinence, urgency, and frequency. With long-term use, the adverse reactions, including constipation and dry mouth, only manifested in a few patients (25). Although the therapeutic effects of M3R inhibition will require further investigation in clinical trials, our preclinical study provides a rationale to evaluate the short-term use of darifenacin for the prevention of post–endoscopic retrograde cholangiopancreatography pancreatitis or treatment in the early stage of AP.

In summary, the activation of M3Rs in the pancreas may provide a clinically relevant model for both AP and CP. M3R may be a promising target for pancreatitis interventions.

## Methods

A detailed description of materials and methods is provided in the Supplemental Methods.

### Mice and pancreatitis induction.

LSL-hM3R (also known as R26-LSL-Gq-DREADD, https://www.jax.org/strain/026220) and LSL-hM4R (also known as R26-LSL-Gi-DREADD, https://www.jax.org/strain/026219) were purchased from The Jackson Laboratory (17). Pdx1-Cre (strain 01XL5) was obtained from the NIH National Cancer Institute Mouse Repository (https://frederick.cancer.gov/science/technology/mouserepository/MouseModels/StrainDetails.aspx?StrainNum=01XL5g=Cre). BAC-Ela-Cre^ERT^ was developed in our laboratory and is available at The Jackson Laboratory (https://www.jax.org/strain/025736) (18). Double-transgenic mice, LSL-hM3R/BAC-Elastase-Cre^ERT^ (hM3/BAC) or LSL-hM3R/Pdx1-Cre (hM3/Pdx1), were developed for the pancreas-specific expression of a mutant human M3R, which lost the ability to respond to the natural ligand acetylcholine but gained the ability to respond to an inert compound (CNO) with nanomolar potencies (17). LSL-hM4R (a DREADD mutant Gi protein–coupled M4R) mice expressing a mutant human M4R were used as a control (17). Tamoxifen (50 mg/kg/d, oral) was given for 3 consecutive days to induce the pancreatic acinar cell–specific expression of the mutant M3R within hM3/BAC mice. In all experiments involving tamoxifen, control groups were also treated with the same dose of tamoxifen. For AP induction, mice were injected with CNO (various doses, ip) or 8 times with cerulein (100 μg/kg/h, ip). Pancreata were harvested 24 hours after the first CNO or cerulein injection. For CP study, ip injections of CNO (6 mg/kg, once daily) or cerulein (100 μg/kg/h, 8 times a day) were repeated 3 times every other day during the first week. Mice were sacrificed at 4 weeks to harvest pancreata. Histology, serum amylase levels, and other pancreatitis parameters were evaluated. Littermate mice were used as controls.

### Amylase activity.

Amylase activity was measured using the Phadebas test as recommended (Pharmacia Diagnostics).

### Histology and immunohistochemistry.

The pancreatic tissues were fixed in a 10% buffered formalin solution for 24 hours, embedded in paraffin, and sectioned for HE, Sirius red, or immunohistochemistry staining. Quantification of the percentage of positive cells was calculated using ImageScope software. Antibodies used in the study are listed in Supplemental Methods.

### Statistics.

All data were expressed as mean ± SEM from experiments. Statistical analysis was performed using GraphPad Prism 7. Comparisons between 2 or more groups were performed using 2-tailed Student’s *t* test or 2-way ANOVA, respectively. A *P* value of less than 0.05 was considered statistically significant.

### Study approval.

The animal experiments were approved by the Institutional Animal Care and Use Committees of Mayo Clinic.

## Author contributions

J Wan and J Wang participated in concept design, performed most of the experiments and data analysis, and drafted the manuscript. J Wan initiated the study and so is listed first, and J Wang was involved in substantial revision. LEW and DIY performed intracellular calcium and trypsinogen activation assays. FG, JC, XZ, ANH, and BHE performed part of the experiments. BH provided fresh human pancreatic acinar cell samples. OHW, DM, and YW were involved in the extensive revision and manuscript preparation. YB and BJ supervised the study, finalized the manuscript, and secured the funding support.

## Supplementary Material

Supplemental data

## Version 1 07/27/2021

In-Press Preview

## Version 2 09/08/2021

Electronic publication
