# Supplementary figures and images for: Pancreas-specific CHRM3 activation causes pancreatitis in mice
Source: JCI Insight. 2021 Sep 8;6(17):e132585. doi: 10.1172/jci.insight.132585 (PMC8492327; doi:10.1172/jci.insight.132585)

# Suppl Fig 1

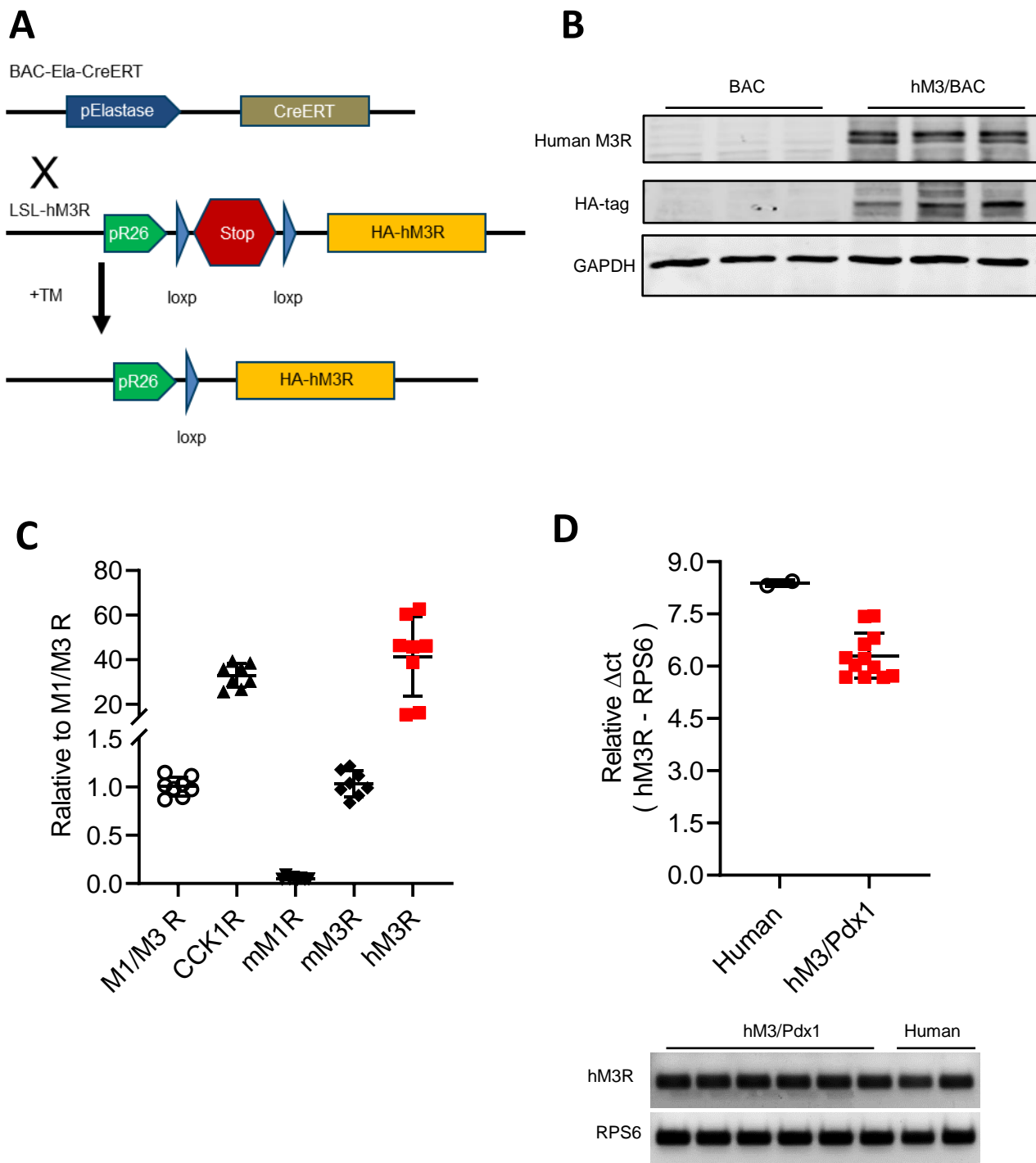

# Suppl Fig 2

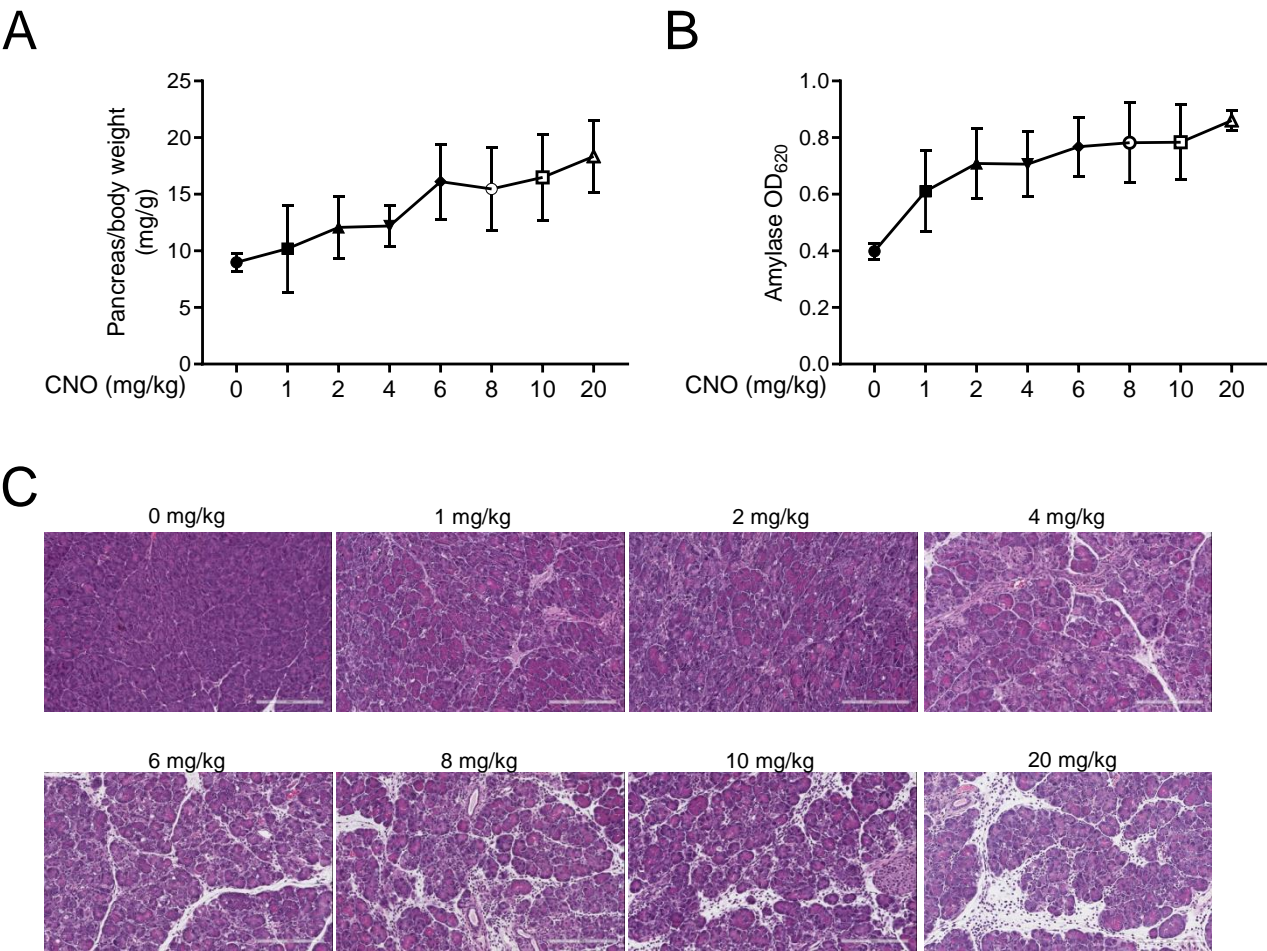

# Suppl Fig 3

A

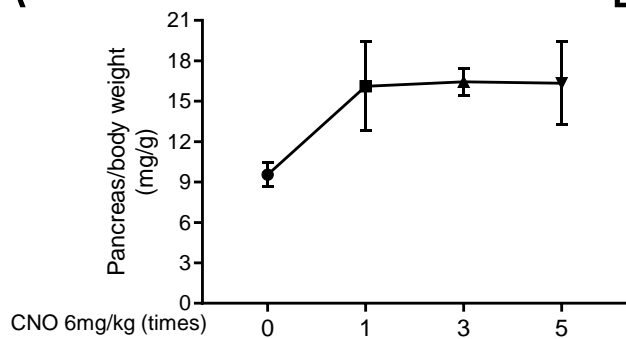

B

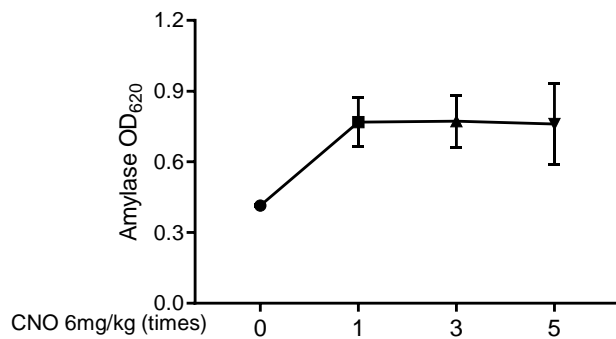

C

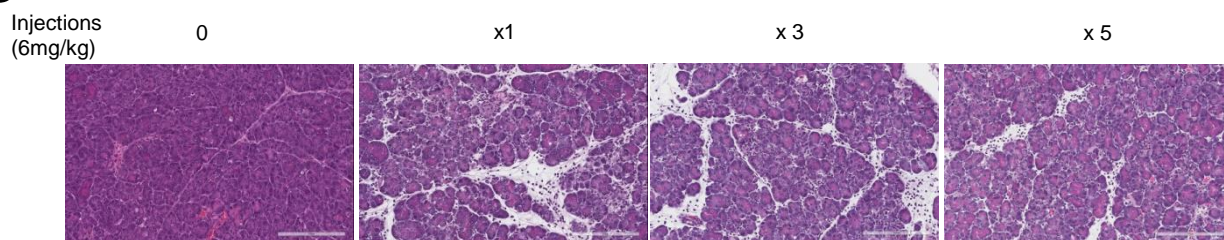

D

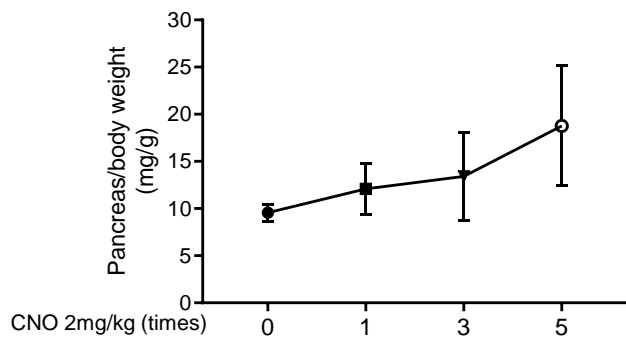

E

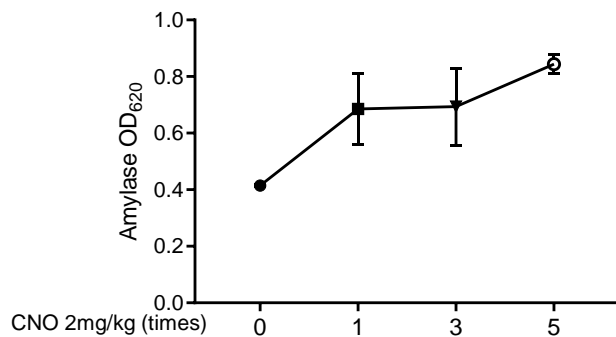

F

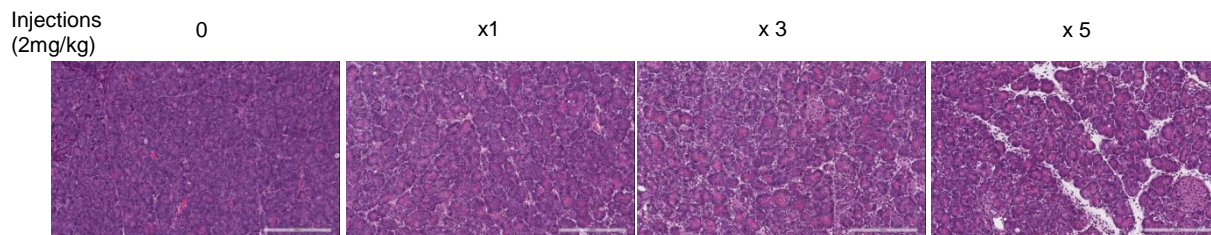

Suppl Fig 4.

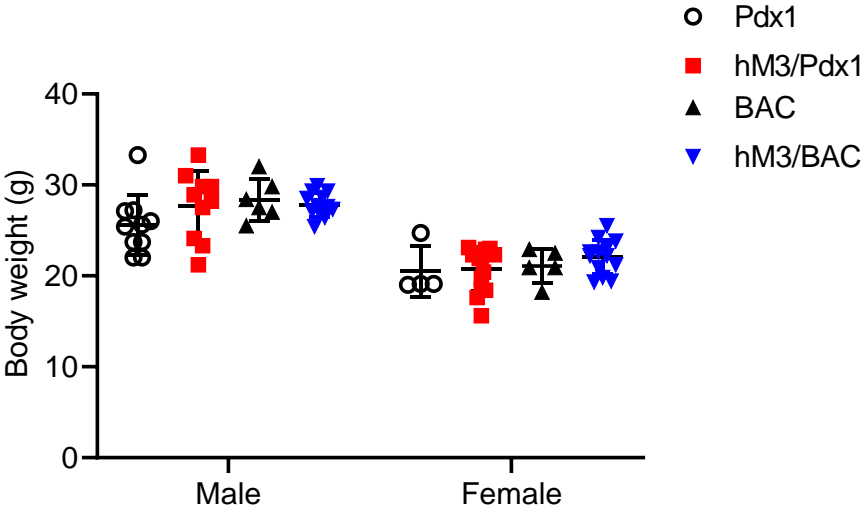

## Suppl Fig 5.

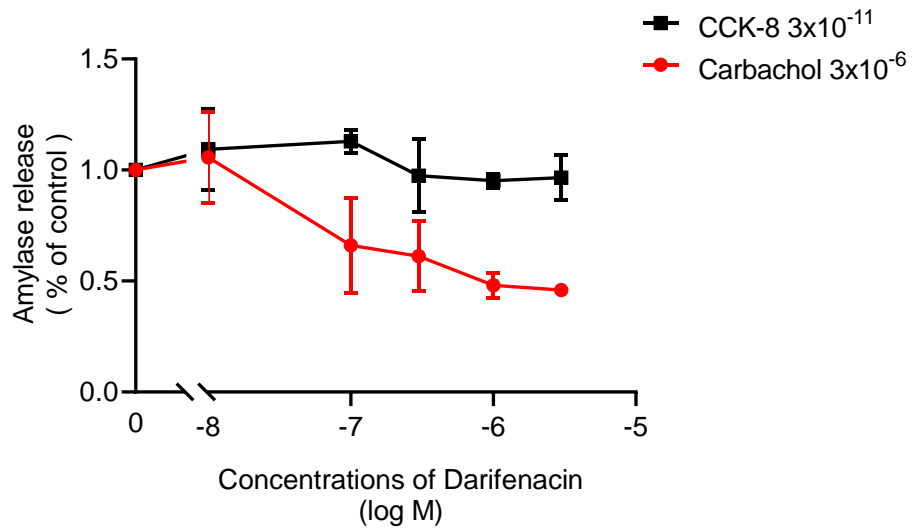

Suppl Fig 6.

A

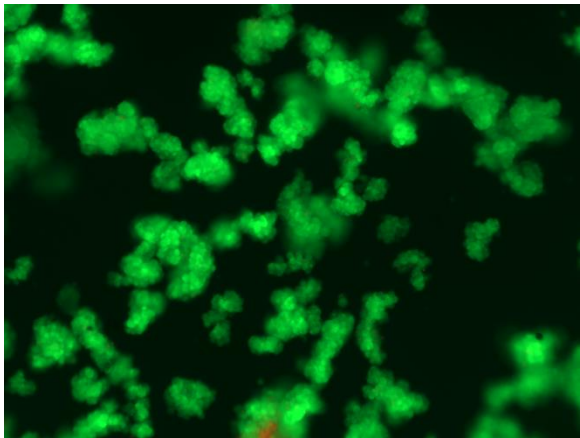

B

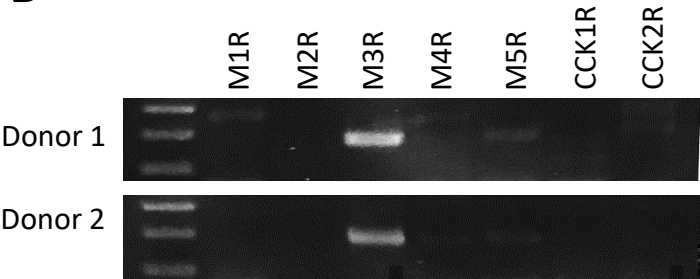

Supplement: Supplemental data [file jciinsight-6-132585-s018.pdf]
